# LB6. Asymptomatic Infection and Duration of Viral Shedding in Symptomatic Breakthrough Infections in a Phase 3 Study of AZD1222 (ChAdOx1 nCoV-19)

**DOI:** 10.1093/ofid/ofab466.1637

**Published:** 2021-12-04

**Authors:** Magdalena Sobieszczyk, Ann R Falsey, Merlin L Robb, Hong-Van Tieu, Julie McElrath, Lawrence Corey, Kathleen Neuzil, Tina Tong, Margaret Brewinski Isaacs, Jill Maaske, Brett Jepson, Stephanie Sproule, Elizabeth Kelly

**Affiliations:** 1 Columbia University Irving Medical Center, New York, NY; 2 University of Rochester, Rochester, New York; 3 Walter Reed Army Institute of Research, Silver Spring, MD, Silver Spring, Maryland; 4 Columbia University Irving Medical Center and New York-Presbyterian Hospital, New York, NY and Laboratory of Infectious Disease Prevention, Lindsley F. Kimball Research Institute, New York Blood Center, New York, NY, New York, New York; 5 Fred Hutchinson Cancer Research Center, Seattle, WA; 6 University of Maryland School of Medicine, Baltimore, Maryland; 7 National Institute of Allergy and Infectious Diseases, National Institutes of Health, Bethesda, MD, Bethesda, Maryland; 8 AstraZeneca Pharmaceuticals LP, Gaithersburg, MD, Gaithersburg, Maryland; 9 AstraZeneca Pharmaceuticals LP, Gaithersburg, MD and Cytel, Inc., Cambridge, MA, Bethesda, Maryland

## Abstract

**Background:**

SARS-CoV-2 vaccine efficacy (VE) against asymptomatic infection and impact on viral shedding during breakthrough infections have critical implications for pandemic control. AZD1222 (ChAdOx1 nCoV-19; 2 doses, 4 weeks apart) demonstrated VE of 74.0% (95% CI 65.3, 80.5) against the primary endpoint of symptomatic RT-PCR-confirmed COVID-19 and safety in a Phase 3, 2:1 randomized, placebo-controlled study in the US, Chile and Peru (n=32,451). Here we present exploratory analyses on asymptomatic infections determined by nucleocapsid (N) seroconversion and time to viral clearance in participants with symptomatic infections determined by N seroconversion (primary data cut, March 5, 2021).

**Methods:**

N seroconversion was assessed at all scheduled and illness visits in the fully vaccinated analysis set (Table). In this analysis, symptomatic infections are defined as N seroconversion ≥ 15 days post second dose in participants who attended an illness visit with ≥ 1 qualifying COVID-19 symptom and had ≥ 1 positive RT-PCR result for SARS-CoV-2. Asymptomatic infections are defined as N seroconversion ≥ 15 days post second dose in participants who did not meet the criteria for symptomatic infections. In participants with symptomatic infections, viral shedding in saliva was assessed for 28 days and cumulative incidence of viral clearance was determined.

Table. AZD1222 VE against symptomatic and potentially asymptomatic SARS-CoV-2 infections as determined by N seroconversion

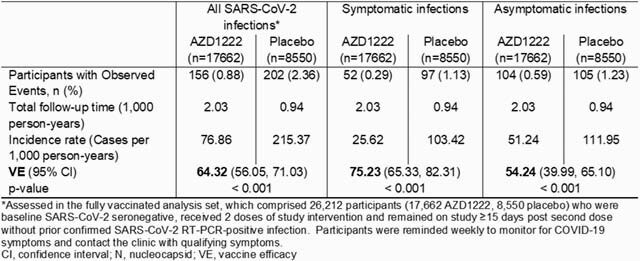

**Results:**

Overall, 358 participants had SARS-CoV-2 infections as determined by N seroconversion (Table). Incidences per 1000 person-years of symptomatic infections were 25.62 for AZD1222 vs 103.42 for placebo (VE 75.23%; 95% CI 65.33, 82.31) and of asymptomatic infections were 51.24 vs 111.95 (VE 54.24%; 95% CI 39.99, 65.10) (Table). Sensitivity analyses for N seroconversion using the primary endpoint and CDC criteria for defining symptomatic/asymptomatic status were supportive. Median time to viral clearance in saliva in participants with symptomatic infections was 11 days (AZD1222, n=52) vs 16 days (placebo, n=92) (Figure).

Figure. Viral clearance in saliva samples in participants with symptomatic infections as determined by N seroconversion

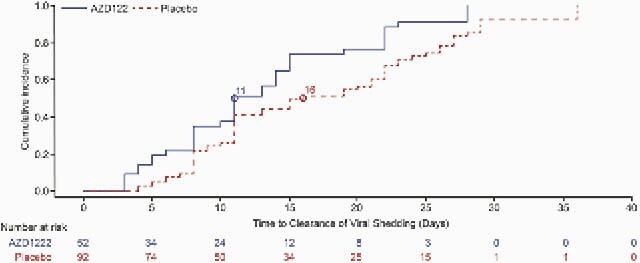

**Conclusion:**

AZD1222 resulted in lower yet meaningful VE against asymptomatic compared to symptomatic infections, as determined by N seroconversion, and shortened viral shedding in symptomatic SARS-CoV-2 breakthrough infections vs placebo, highlighting its potential contribution to reducing viral transmission.

Funding Statement

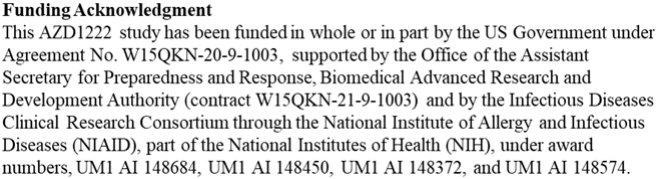

**Disclosures:**

**Ann R. Falsey, MD**, AstraZeneca (Individual(s) Involved: Self): Grant/Research Support; BioFire Diagnostics (Individual(s) Involved: Self): Grant/Research Support; Janssen (Individual(s) Involved: Self): Grant/Research Support; Merck, Sharpe and Dohme (Individual(s) Involved: Self): Grant/Research Support; Novavax (Individual(s) Involved: Self): Other Financial or Material Support, Paid DSMB member; Pfizer (Individual(s) Involved: Self): Grant/Research Support **Merlin L. Robb, M.D.**, **Henry M. Jackson Foundation** (Other Financial or Material Support, This work was conducted under a funding agreement with the USG through an INteragency Personnel Agreement) **Hong-Van Tieu, M.D, M.S.**, **National Institutes of Health** (Grant/Research Support) **Lawrence Corey, MD**, **NIH** (Grant/Research Support) **Kathleen Neuzil, MD, MPH**, **NIH** (Grant/Research Support, Other Financial or Material Support, Dr. Neuzil is the CoVPN co-chair with testing of COVID-19 vaccines with salary support from NIH.)**Pfizer** (Grant/Research Support) **Jill Maaske, M.D.**, **AstraZeneca** (Employee, Shareholder) **Brett Jepson, n/a**, **AstraZeneca** (Other Financial or Material Support, On assignment to AstraZeneca)**Cytel, Inc.** (Employee) **Stephanie Sproule, n/a**, **AstraZeneca** (Other Financial or Material Support, Fees for statistical consulting.) **Elizabeth Kelly, n/a**, **AstraZeneca** (Employee, Shareholder)

